# Combined versus standalone XEN45 Gel Stent implantation in either phakic or pseudophakic patients: a case-matched study

**DOI:** 10.1007/s00417-023-06283-y

**Published:** 2023-11-11

**Authors:** David Kiessling, Corinna Rennings, Matthias Hild, Alexandra Lappas, Thomas Stefan Dietlein, Gernot Franz Roessler, Randolf Alexander Widder

**Affiliations:** 1 Department of Ophthalmology, St. Martinus-Krankenhaus Düsseldorf, Gladbacher Str. 26, 40219 Düsseldorf, Germany; 2 Department of Ophthalmology, University Hospital of Cologne, Cologne, Germany; 3 Department of Ophthalmology, RWTH Aachen, Aachen, Germany

**Keywords:** Glaucoma incisional surgery, MIGS, XEN, Microstent, Cataract surgery, Open-angle glaucoma

## Abstract

**Purpose:**

To determine differing outcomes among either phakic or pseudophakic patients who received standalone XEN45 Gel Stent (Allergan, an AbbVie Company, CA, USA) implantation and patients who underwent combined surgery with phacoemulsification.

**Methods:**

This retrospective single-center study involved 180 eyes of 180 participants who underwent XEN45 Gel Stent implantation, of which 60 eyes received combined surgery with phacoemulsification (combined group). Standalone stent implantation was performed on 60 phakic (phakic group) and on 60 pseudophakic eyes (pseudophakic group). The groups were matched in a ratio of 1:1:1 based on multiple criteria. Successful surgery was defined by three scores: IOP at the longest follow-up of < 21 mmHg (Score A) or < 18 mmHg (Score B) and an IOP reduction > 20% or IOP ≤ 15 mmHg and an IOP reduction ≥ 40% (Score C). In all scores, one open conjunctival revision was allowed, and additional repeat surgery was considered a failure.

**Results:**

After an average follow-up time interval of 20.6 ± 12.6 months, there was a mean IOP-reduction by 37% among the entire cohort. Comparative analysis between the three groups did not show significant differences regarding postoperative IOP, postoperative medication score, side effects, revision rate, repeat surgery rate or success rate. A dysfunctional stent was detected in eight eyes (4%) during open conjunctival revision in 76 eyes.

**Conclusion:**

The clinical endpoints investigated did not differ significantly among either phakic or pseudophakic patients who received standalone stent implantation and patients who underwent combined surgery. However mean latency between primary stent implantation and first revision surgery after combined surgery was markedly shorter.

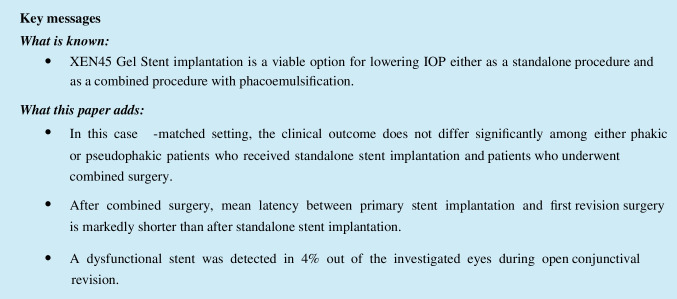

## Introduction

For the past decades, traditional filtering surgery, such as trabeculectomy or the implantation of drainage devices, has been one of the main options for treating uncontrolled glaucoma.

More recently, microinvasive glaucoma surgery (MIGS) has been used in routine clinical practice, in addition to established procedures. XEN45 Gel Stent implantation (Allergan, an AbbVie Company, Dublin, CA, USA), being just one of them, is a subconjunctival bleb-forming procedure, which pursues an ab-interno approach. Primary implantation is carried out via the anterior chamber through the trabecular meshwork and the scleral wall; therefore, it does not require penetration of the conjunctiva. The implant has a small lumen, which assures moderate drainage of aqueous humor into the subconjunctival space with fewer side effects such as hypotony. The favorable safety profile, along with the promising potential for lowering IOP, has been reported in several studies [1–5].

However, secondary interventions are frequently needed due to postoperative scarring of the conjunctiva. Several studies have reported that combined stent implantation with phacoemulsification results in higher revision rates than the standalone procedure [1, 6–9]. This finding supports the question whether the phakic status exerts an influence on the overall outcome of XEN45 Gel Stent implantation.

To date, there has been no case-matched investigation that compares multiple clinical endpoints between subgroups of either phakic or pseudophakic patients who underwent the standalone procedure and patients who received combined stent implantation with phacoemulsification.

For this reason, we compared postoperative IOP, postoperative medication score, side effects, revision rate, repeat surgery rate, and success rate using different clinical scores between the three groups with different phakic statuses.

## Methods

### Study design and patients

This was a single-center retrospective study based on data acquired from the Department of Ophthalmology, St. Martinus − Krankenhaus Düsseldorf, Germany. The study included phakic (phakic group) and pseudophakic patients (pseudophakic group) who underwent XEN45 Gel Stent implantation as a standalone procedure and phakic patients who underwent combined surgery with phacoemulsification (combined group) between 2015 and 2020. The inclusion criteria were as follows: primary open-angle glaucoma (POAG), an available baseline, and a postoperative follow-up IOP value of at least 6 months. Eyes with concomitant diseases, such as neovascular glaucoma, exfoliation glaucoma, uveitis, and vitrectomized eyes, were excluded.

The three groups were matched in a ratio of 1:1:1 via a case–control matching procedure. The following criteria were taken into account: preoperative and maximum intraocular pressure, preoperative medication score, cup/disc ratio, and follow-up time. Additional parameters, such as type of glaucoma, ethnicity, best-corrected visual acuity (BCVA) at baseline, and age, were extracted from the patients’ files. If both eyes of a patient were eligible, eyes with a longer follow-up were included in the study.

IOP was assessed using Goldmann applanation tonometry. A maximum of three IOP measurements were collected before the date of surgery and averaged to evaluate the baseline IOP. During follow-up admissions to the outpatient clinic, a singular IOP measurement was carried out in the morning using Goldmann applanation tonometry by a non-masked observer. The medication score comprised the amount of IOP-lowering medication classes applied at baseline and follow-up. BCVA was measured using standard Snellen charts.

### Surgical technique

Implantation of the XEN45 Gel Stent was performed as described previously [1]. First, a 0.1 ml dose of mitomycin C (0.1 mg/ml) was injected under the conjunctiva of the upper nasal quadrant, at a distance of 6 mm from the limbus. After temporal paracentesis and paracentesis in the 5 or 7 o’clock position and stabilization of the anterior chamber using a viscoelastic substance, the stent was placed via its injector device. The apex of the injector was driven through the trabecular meshwork and sclera, with a distance of 3 mm from the limbus. The stent was then injected under the conjunctiva, and the injector was removed. The position of the stent was confirmed by gonioscopy, and the viscoelastic substance was removed from the anterior chamber by irrigation.

When XEN45 Gel Stent implantation was combined with phacoemulsification, a standard 2.8 mm clear cornea incision was made in the temporal cornea after subconjunctival injection of mitomycin C. Two paracenteses were positioned at a 90° angle to the tunnel, following bimanual phacoemulsification with posterior chamber and in-the-bag intraocular lens (IOL) implantation. Subsequently, stent implantation was performed as previously described.

The surgery aimed to regulate the IOP without antiglaucomatous medication. Therefore, all patients without a sufficiently reduced IOP postoperatively underwent surgical revision. Instead of needling, we performed an open conjunctival approach according to the standard operating procedure of our department, which we have described in detail before [1, 6]. Briefly, the conjunctiva was incised at the limbus, and the stent was prepared. After the removal of scar tissues, the conjunctiva was refixated at the limbus with two absorbable 9.0 sutures.

Standardized postoperative topical therapy was applied, including antibiotic ointment (Floxal AS, Bausch & Lomb, Frankfurt, Germany) three times a day and steroid ointment (Ultracortenol AS, Agepha, Senec, Slovakia) three times a day for 4 weeks. Previous topical anti-glaucomatous medications were discontinued.

### Outcome measurement

The primary clinical endpoints were changes in IOP and medication scores at the longest follow-up examination. The outcomes were further defined as success or failure by three separate scores: according to Score A and Score B, an absolute IOP of < 21 mmHg (Score A) or < 18 mmHg (Score B) at the follow-up examination and a postoperative IOP reduction of > 20%, and no repeat surgery qualified for success. Two scores were chosen according to the Tube versus Trabeculectomy Study [10]. The criteria for Score C were an absolute IOP of ≤ 15 mmHg and a postoperative IOP reduction of ≥ 40%, and no repeat surgery according to the criteria of the World Glaucoma Association [11]. In all scores, one open conjunctival revision was allowed, additional repeat surgery counted as a failure.

### Statistical analysis

Statistical analyses were performed using SPSS (version 24.0; IBM Corp. Armonk, NY, USA) and the statistical programming language R V3.2.2 (R Foundation for Statistical Computing, Vienna, Austria). The comparison between the outcome measurements in the combined, phakic, and pseudophakic groups was carried out using one-way analysis of variance (ANOVA). Statistically significant ANOVA results were further investigated using the Bonferroni test for post hoc multiple comparisons.

Furthermore, the log-rank test was carried out, as well as visualization using Kaplan − Meier curves. Subanalyses were carried out for each follow-up timeframe at 6 months, 12 months, 24 months, 36 months, and 48 months respectively. The resulting threshold for statistical significance was set at *p* < 0.05.

Power calculation was conducted using Minitab Statistical 18.1 (Minitab Inc., State College, PA, USA).

### Ethics and statistics

The study and data collection were conducted with the approval of the institutional review board (Ethik und Kommission Klinische Studien, Dernbacher Gruppe Katharina Kasper, Germany). The tenets of the Declaration of Helsinki were regarded.

## Results

A total of 781 eyes of 609 patients were identified in our database, with 614 eyes having received standalone XEN45 Gel Stent implantation, of which 491 eyes were pseudophakic, and 123 eyes were phakic. Meanwhile, 167 eyes underwent combined stent implantation with cataract surgery. The inclusion criteria were met by 144, 69 and 104 eyes in the pseudophakic, phakic and combined groups, respectively. After matching, 60 eyes of 60 patients remained in each group. Baseline data are summarized in Table 1.

**Table 1:** Baseline data

	#1 Combined with phaco *n* = 60	#2 Standalone phakic *n* = 60	#3 Standalone pseudophakic *n* = 60	ANOVA *p*	*p* #1 vs. #2	Bonferroni *p* #2 vs. #3	*p* #1 vs. #3
Age (years)	72.3 ± 11.0	60.5 ± 12.0	72.4 ± 7.2	< 0.05	< 0.05	< 0.05	> 0.99
Caucasian	60/60	60/60	60/60	> 0.99	> 0.99	> 0.99	> 0.99
Maximum preoperative IOP (mmHg)	31.4 ± 9.8	32.0 ± 10.2	31.3 ± 6.2	0.91	> 0.99	> 0.99	> 0.99
Actual preoperative IOP (mmHg)	23.5 ± 5.7	23.4 ± 5.1	23.4 ± 4.6	> 0.99	> 0.99	> 0.99	> 0.99
Medication score initial	2.3 ± 1.2	2.5 ± 1.1	2.6 ± 1.1	0.35	> 0.99	> 0.99	0.44
Baseline C/D ratio	0.7 ± 2.1	0.7 ± 0.2	0.8 ± 0.2	0.08	0.17	> 0.99	0.15
Follow-up time (months)	20.8 ± 13.6	20.6 ± 14.5	20.5 ± 9.5	0.99	> 0.99	> 0.99	> 0.99
Baseline BCVA (logMAR)	0.4 ± 0.4	0.2 ± 0.3	0.4 ± 0.5	< 0.05	< 0.05	< 0.05	> 0.99
Previous glaucoma surgery							
Trabeculectomy, n (%)	3 (5.0)	5 (8.3)	8 (13.3)	0.28	> 0.99	> 0.99	0.33
Deep Sclerectomy, n (%)	1 (1.7)	0	3 (5.0)	0.17	> 0.99	0.19	0.65
Trabectome surgery, n (%)	0	2 (3.3)	10 (16.6)	< 0.05	> 0.99	< 0.05	< 0.05
SLT, n (%)	6 (10.0)	13 (21.7)	12 (20.0)	0.19	0.28	> 0.99	0.45

*BCVA* best-corrected visual acuity; *C/D* Cup/Disc; *IOP* intraocular pressure; *SLT* selective laser trabeculoplasty

The threshold for statistical significance is *p* < 0.05

The spectrum of prior eye surgery comprised of trabeculectomy (n = 16), deep sclerectomy (n = 4) Trabectome surgery (n = 14), and selective laser trabeculoplasty (n = 38). A subanalysis of prior glaucoma surgery among the three groups taking the most invasive procedure into consideration is given in Table 1. The number of filtering surgery, as well as the number of selective laser trabeculoplasty did not differ significantly.

After an average follow-up time interval of 21 ± 12.6 months, there was a mean IOP-reduction of 37%, from 23.4 ± 5.1 to 14.8 ± 5.1 mmHg, and a mean reduction of the medication score of 88%, from 2.5 ± 1.1 to 0.3 ± 0.8 mmHg among the entire cohort of 180 patients (Table 2).

**Table 2:** Intraocular pressure and medication score at baseline and the longest follow-up

	All *n* = 180	Combined with phaco *n* = 60	Standalone phakic *n* = 60	Standalone pseudophakic *n* = 60	*p*
IOP preoperative (mmHg)	23.4 ± 5.1	23.5 ± 5.7	23.4 ± 5.1	23.4 ± 4.6	> *0.99*
IOP postoperative (mmHg)	14.8 ± 5.1	14.6 ± 5.3	14.7 ± 5.5	15.2 ± 4.4	*0.78*
Medication score preoperative	2.5 ± 1.1	2.3 ± 1.2	2.5 ± 1.1	2.6 ± 1.1	*0.35*
Medication score postoperative	0.3 ± 0.7	0.3 ± 0.7	0.3 ± 0.7	0.2 ± 0.6	*0.72*
Revision	76/180 (42%)	30/60 (50%)	21/60 (35%)	25/60 (42%)	*0.25*
Repeat surgery	20/180 (11%)	5/60 (8%)	5/60 (8%)	10/60 (17%)	*0.17*

*IOP* intraocular pressure

On the first postoperative day IOP was significantly lower in the pseudophakic group than in the phakic and combined group (p = 0.008). Throughout the follow-up timeframes at 1 month, 3 months, 6 months, 12 months, 24 months, 36 months, and 48 months postoperative IOP values remained stable at low levels, all three groups did not show significant differences during follow-up (Table 3).

**Table 3:** Mean IOP (mmHg) at baseline and during follow-up

	*n*	All	#1 Combined with phaco	#2 Standalone phakic	#3 Standalone pseudophakic	*p*
Baseline	180	23.4 ± 5.1	23.5 ± 5.7	23.4 ± 5.1	23.4 ± 4.6	> 0.99
Postop	180	9.7 ± 5.1	11.0 ± 4.4	10.0 ± 6.0	8.2 ± 4.6	< 0.01
1 month	136	14.9 ± 6.7	16.5 ± 7.7	15.0 ± 6.4	13.1 ± 5.5	0.05
3 months	94	15.5 ± 5.4	16.2 ± 6.5	15.0 ± 5.3	15.6 ± 4.4	0.64
6 months	123	14.4 ± 3.8	13.8 ± 4.0	14.8 ± 3.5	14.5 ± 3.8	0.47
9 months	46	13.5 ± 3.5	12.9 ± 2.4	13.4 ± 3.3	14.4 ± 5.1	0.60
12 months	109	14.7 ± 4.4	14.7 ± 4.9	14.4 ± 3.9	15.0 ± 4.3	0.88
18 months	52	14.2 ± 3.5	13.8 ± 3.3	13.8 ± 2.0	14.6 ± 4.2	0.72
24 months	65	13.6 ± 3.6	12.7 ± 2.9	13.2 ± 4.2	14.9 ± 3.5	0.11
36 months	22	14.6 ± 6.5	12.5 ± 2.8	17.2 ± 9.4	13.2 ± 1.3	0.29
48 months	12	15.3 ± 5.8	14.6 ± 4.7	15.8 ± 7.4	15.0	0.95
60 months	3	22.3 ± 14.3	24.0 ± 19.8	19.0		0.87

*IOP* intraocular pressure

The threshold for statistical significance is *p* < 0.05

**Fig. 1:**
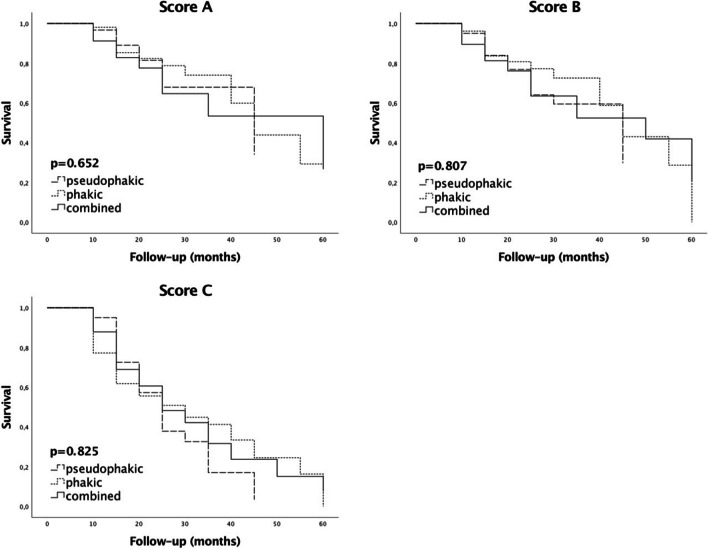
Kaplan − Meier survival curves comparing the success rates in the combined, phakic, and pseudophakic groups after XEN45 Gel Stent implantation. Score A: IOP at follow-up < 21 mmHg, IOP reduction > 20%, one revision surgery allowed, no repeat surgery. Score B: IOP at follow-up < 18 mmHg, IOP reduction > 20%, one revision surgery allowed, no repeat surgery. Score C: IOP at follow-up ≤ 15 mmHg, IOP reduction ≥ 40%, one revision surgery allowed, no repeat surgery

**Fig. 2:**
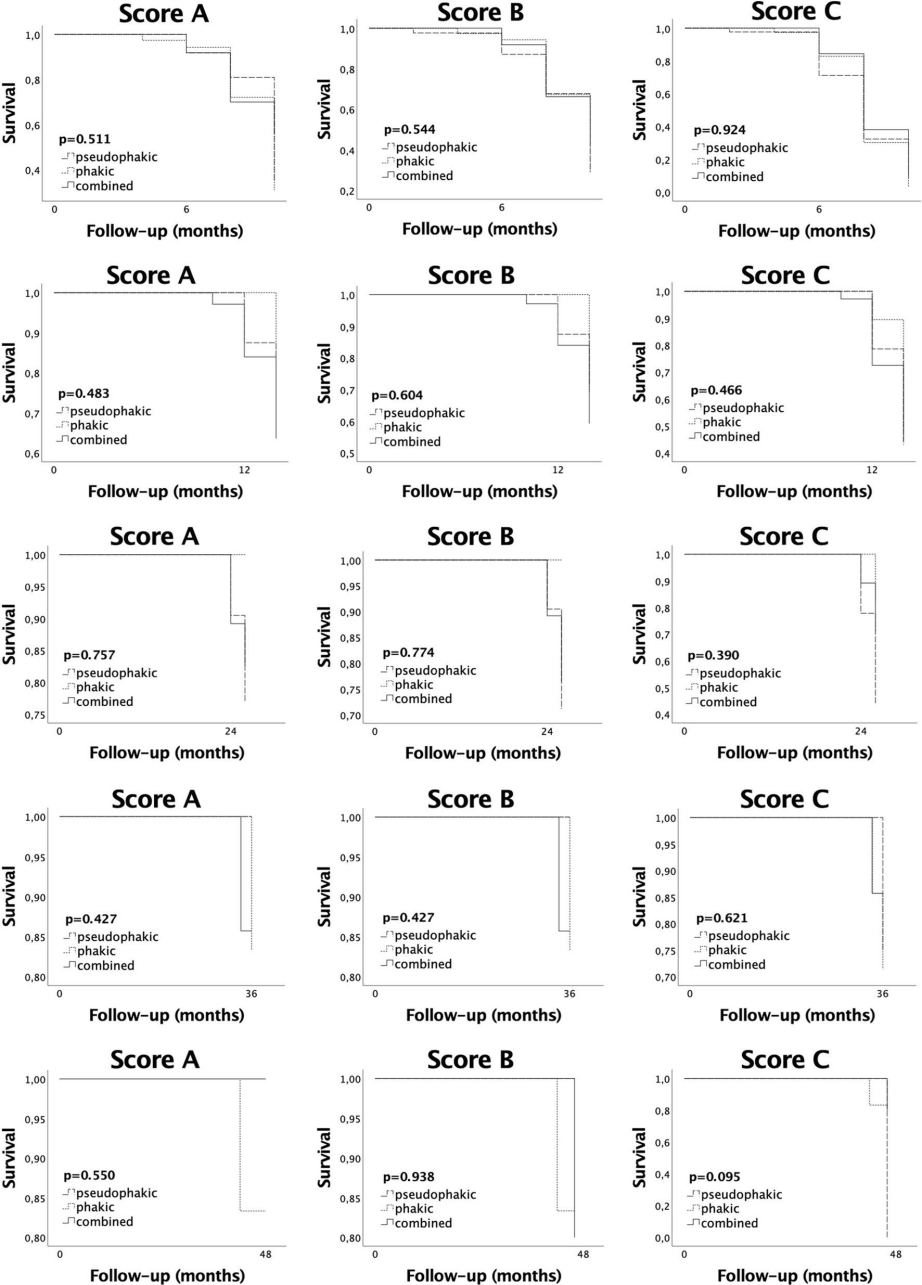
Kaplan − Meier survival curves within the follow-up timeframes at 6 months, 12 months, 24 months, 36 months, and 48 months. Score A: IOP at follow-up < 21 mmHg, IOP reduction > 20%, one revision surgery allowed, no repeat surgery. Score B: IOP at follow-up < 18 mmHg, IOP reduction > 20%, one revision surgery allowed, no repeat surgery. Score C: IOP at follow-up ≤ 15 mmHg, IOP reduction ≥ 40%, one revision surgery allowed, no repeat surgery

Comparative analysis between the combined, phakic, and pseudophakic groups did not show a significant difference in postoperative IOP, postoperative medication score, revision rate, repeat surgery rate, or success rate (Table 2, Fig. 1). The respective survival rates withing each follow-up timeframe show a similar trend (Fig. 2).

A total of 76 eyes underwent one open conjunctival revision, of which 20 eyes required a second surgical revision using the same technique. One of these eyes underwent an additional trabeculectomy at a later time point. In eight revised eyes (11%), the intraoperative preparation of the stent showed a functional deficit, and the stent was replaced in the same surgical session. The mean latency between primary stent implantation and the first revision surgery was 8 ± 11.4 months in the combined group, 10 ± 10.0 months in the phakic group, and 13 ± 10.7 months in the pseudophakic group.

Side effects included hyphaema (n = 12), self-limited choroidal effusion (n = 6), and a self-limited shallow anterior chamber (n = 2) due to transient hypotony and stent exposure (n = 2). Stent exposure required open conjunctival revision and was counted as a repeat surgery. No severe side effects, such as retinal detachment, leakage, blebitis, or endophthalmitis, were observed (Table 4).

**Table 4:** Complications

	Combined with phaco *n* = 60	Standalone phakic *n* = 60	Standalone pseudophakic *n* = 60	*p*
Hyphaema	3	5	4	0.77
Shallow anterior chamber	1	1	0	0.61
Stent exposure	1	2	0	0.37
Chorioidal effusion	2	2	2	> 0.99
Macular edema, retinal detachment, leakage, blebitis, endophthalmitis	0	0	0	

The threshold for statistical significance is *p* < 0.05

The Kaplan − Meier curves comparing the success rates of the two groups according to the three scores applied displayed the corresponding trends, which remained parallel throughout (Fig. 1).

Moreover, empirical power calculation for the size of the study was performed according to the dichotomous endpoint of Score A. The success rates were 78% in the combined group, 75% in the phakic group and 70% in the pseudophakic group. Our analysis (n = 180) revealed a post-hoc power of 75% to detect a difference of ± 20% between the three groups. The power was 26% for a corresponding ± 10%-difference and 10% for a ± 5%-difference.

## Discussion

We found that there were no significantly different outcomes among the groups of either phakic or pseudophakic patients who received standalone stent implantation and those who underwent the combined procedure with phacoemulsification concerning the clinical endpoints investigated.

Furthermore, our study provided further evidence that XEN45 Gel Stent implantation effectively reduces both IOP and medication scores while retaining a favorable safety profile, which has been reported in previous studies [1, 3, 4]. Among our entire cohort, a reduction in the mean IOP of 37% was observed, a postoperative IOP of 14.8 ± 5.1 mmHg and a postoperative medication score of 0.3 ± 0.8 mmHg. Open conjunctival revision surgery was performed in 42% of the patients, which being with the respective rates calculated in previous studies [3, 5, 12, 13]. Repeat surgery was necessary for 11% of all patients.

Although the revision rate was higher in the combined group than in both groups who underwent standalone surgery, there was no statistically significant difference determined within the context of our case-matched study. However, the mean latency between primary stent implantation and first revision surgery in the former group was markedly shorter. This might explain the findings of Widder et al., who reported that combined surgery resulted in a higher revision rate than standalone surgery in a study with a shorter follow-up period [1]. In the present study, we were able to provide data for an extended mean follow-up, during which these rates ultimately did not differ significantly.

Our results are consistent with the findings of a meta-analysis on XEN45 Gel Stent implantation with and without cataract surgery [14]. Wang et al. observed that standalone surgery leads to better results than combined stent implantation with phacoemulsification only in the early postoperative phase and that IOP-lowering efficiency was equalized between the groups after several months. The authors hypothesized that, while performing an additional cataract extraction, the increased use of viscoelastic substances leads to more residues, which in turn might affect aqueous flow through the small lumen at an early stage after combined surgery [14].

Combining traditional filtering surgery with phacoemulsification has been the subject of numerous studies. In the context of a meta-analysis, Friedman et al. found evidence that phacotrabeculectomy results in less sufficient IOP reduction than standalone trabeculectomy [15].

It was postulated that combined surgery increases trauma, leading to higher levels of transforming growth factor beta 2 in the aqueous humor and prolonged alteration of the blood-aqueous barrier [16, 17]. Assuming that either of these mechanisms apply to combined XEN45 Gel Stent implantation with cataract surgery, an alteration of molecular properties in the anterior chamber following combined surgery might contribute to a less predictable response of IOP as a result of postoperative inflammation.

Limitations of the study were the retrospective study design as well as the inherent limited sample size. It can be argued that a further limitation of the study might result from the inclusion of patients with previous glaucoma surgery namely trabeculectomy, deep sclerectomy, Trabectome surgery and selective laser trabeculoplasty, because it is widely known that susceptibility to subconjunctival scarring is increased by previous surgeries such as trabeculectomy, cataract surgery, vitrectomy, and keratoplasty, which is of crucial importance for the long-term effectiveness of subsequent filtering surgery [18].

We decided to include these patients for two reasons. First, the idea of the study was to provide case-matched results between the study groups for the first time instead of unmatched patient groups. Including these patients, we were able to provide a number of patients with sufficient statistical power. Secondly and more importantly, we found it reasonable to believe that the inclusion does not affect the study results because in two previous case-matched studies we were able to show that previous Trabectome surgery as well as previous trabeculectomy does not affect the outcome of a XEN45 Gel Stent implantation [19, 20].

Therefore, previous glaucoma surgeries such as trabeculectomy, deep sclerectomy, ab-interno trabeculectomy, and selective laser trabeculoplasty were not considered exclusion criteria.

Notably, age and BCVA were not matched criteria among the three groups. This is because we decided to include a subgroup of patients who were left phakic, who had better vision and were naturally of younger age compared to the group who had received combined cataract surgery or were pseudophakic at the time of stent implantation. We acknowledge that this possibly introduced a selection bias, as young age is associated with a higher susceptibility to subconjunctival scarring [18].

We are aware that the results of the study might not be applicable to surgical settings in all glaucoma centers, because our strategy was to perform an open conjunctival revision instead of a needling to reach an appropriate IOP without additional IOP-lowering drugs. This goal might be discussed as ambitious for a MIGS procedure, but after XEN45 Gel Stent implantation using subconjunctival Mitomycin C a quadrant of conjunctiva is lost for further surgery such as trabeculectomy and therefore we aimed to withdraw all glaucoma medication after surgery. After starting with XEN45 Gel Stent implantation we have made the experience that needling is not as effective as open conjunctival revision in our hands and therefore open conjunctival revision became the standard procedure for uncontrolled IOP after XEN45 Gel Stent implantation in our center. To compare our findings with the results of other studies, we did not count one open conjunctival revision as a failure because usually in other studies needling did not count as a failure and the frequency of reported needlings matches the frequency of open conjunctival revision in our study. This might lead to a further limitation of the study. Assumed that open conjunctival revision leads to better results, this might result in a more favorable outcome compared to other studies using needling as a standard procedure for revision [21]. Considering that our standard operating procedure for revision surgery requires the penetration of the conjunctiva, we reckon that its advantage over trabeculectomy is that it assures a favorable pressure control with fewer side effects such as hypotony.

To date, there have been no data on dysfunctional XEN45 Gel Stents. We found that of all stents that required revision surgery, 11% showed a functional deficit. In contrast to needling, open conjunctival revision surgery allows the surgeon to assess a lack of flow and therefore the need for stent replacement [1, 6, 22].

In summary, we validated that for an average follow-up time interval of 21 ± 12.6 months, clinical endpoints, including postoperative IOP, postoperative medication score, side effects, revision rate, repeat surgery rate, and success rate, did not differ significantly among the groups of either phakic or pseudophakic patients who received standalone stent implantation and those who underwent the combined procedure with phacoemulsification. However, the mean latency between primary stent implantation and the first revision surgery in the latter group was markedly shorter.

## Abbreviations

ANOVA: Analysis of variance
BCVA: Best-corrected visual acuity
C/D: Cup/disc
IOL: Intraocular lens
IOP: Intraocular pressure
MIGS: Microinvasive glaucoma surgery
POAG: Primary open-angle glaucoma
SD: Standard deviation
SLT: Selective laser trabeculoplasty
